# Physician self-reported factors driving clinical decision-making in management of patients with T2D and ASCVD/high risk of ASCVD across the middle East and Africa: a cross-sectional study

**DOI:** 10.3389/fphar.2025.1558515

**Published:** 2025-09-09

**Authors:** Sam Salek, Hani Sabbour, Naji Alamuddin, Fatheya Alawadi, Hessa Alkandari, Wael Almahmeed, Samir H. Assaad-Khalil, Emel Mashaki Ceyhan, Jihad Haddad, Landman Lombard, Mary Ngome, Rayaz A. Malik, Gourav Yadav

**Affiliations:** ^1^ School of Life and Medical Sciences, University of Hertfordshire, Hatfield, United Kingdom; ^2^ Heart, Vascular & Thoracic Institute at Cleveland Clinic Abu Dhabi, Abu Dhabi, United Arab Emirates; ^3^ Royal College of Surgeons in Ireland–Bahrain, King Hamad University Hospital, Adilya, Bahrain; ^4^ Endocrine Department, Dubai Hospital, Dubai Health Authority, Dubai, United Arab Emirates; ^5^ Department of Pediatrics, Farwaniya Hospital, Kuwait City, Kuwait; ^6^ Population Health Department, Dasman Diabetes Institute, Kuwait City, Kuwait; ^7^ Unit of Diabetes, Lipidology & Metabolism; Department of Internal Medicine/Alexandria Faculty of Medicine, Alexandria University, Egypt, Egypt; ^8^ Novo Nordisk A/S, Søborg, Denmark; ^9^ Endocrinology Section, Bader Medical Complex, Amman, Jordan; ^10^ Cape Town Medical Research Centre, Cape Town, South Africa; ^11^ Novo Nordisk Saglik Urunleri Tic Ltd Sti, Istanbul, Türkiye; ^12^ Department of Medicine, Weill Cornell Medicine-Qatar, Education City, Doha, Qatar

**Keywords:** atherosclerotic cardiovascular disease, clinical decision-making, heart disease risk factors, public health systems research, type 2 diabetes

## Abstract

**Aims:**

To explore factors influencing the clinical decision-making of physicians treating patients with type 2 diabetes (T2D) and high risk of atherosclerotic cardiovascular disease (ASCVD) across seven Middle Eastern and African countries.

**Methods:**

Cross-sectional, anonymous online study of self-reported factors driving clinical decision-making for the management of T2D based on agreement with statements using a five-point Likert scale among physicians (n = 385) in Bahrain, Egypt, Jordan, Kuwait, Qatar, South Africa, and UAE between June 13 and October 1, 2022.

**Results:**

From a selection of patient factors, physicians were most likely to agree that treatment adherence/compliance (92%), safety concerns (92%), and impact on health-related quality of life (88%) influenced their decision-making. Most physicians agreed that availability of treatment (87%) was a practice setting factor that influenced their decision-making. The top three physician factors influencing clinical decision-making included continuous medical education (96%), medical knowledge (96%), and international clinical guidelines (95%). Most physicians agreed that improved communication skills of physicians (97%), coaching and question prompts for patients (91%), and patient decision aids (87%) could improve shared decision-making.

**Conclusion:**

Various patient, practice, and physician factors influenced physicians’ management of their patients with T2D. Physicians believed improving their communication skills could improve shared decision-making.

**Clinical Trial Registration:**

The trial is registered with clinicaltrails.gov, Identifier #NCT05317845 (2023-03-28).

## 1 Introduction

The worldwide prevalence of type 2 diabetes (T2D) is increasing at an alarming rate (IDF, 2021). The Global Burden of Disease Study in 2021, established that there were 529 million people living with diabetes worldwide, and the global age-standardised total diabetes prevalence was 6.1% (5.8%–6.5%) (Ong et al., 2023). The highest age-standardised rates were observed in North Africa and the Middle East (9.3% [8.7%–9.9%]) and Oceania (12.3% [11.5%–13.0%]), with the highest age-specific prevalence of diabetes, at 76.1% (73.1%–79.5%) in individuals aged 75–79 years in Qatar (Ong et al., 2023).

Cardiovascular disease, particularly atherosclerotic cardiovascular disease (ASCVD), comprised of coronary heart disease, cerebrovascular disease, and peripheral artery disease, is the most frequent cause of morbidity and mortality in patients with T2D (Gerstein, 2015; Hudspeth, 2018). Cardiovascular outcomes trials have demonstrated the efficacy of sodium-glucose transport protein 2 (SGLT2) inhibitors and glucagon-like peptide-1 receptor agonists (GLP-1 RAs) in reducing risk of adverse cardiovascular events in patients with T2D and high cardiovascular risk (ADA and 9, 2021). Indeed, international guidelines now strongly emphasize the importance of improving cardiovascular outcomes in patients with T2D (Marx et al., 2023; Visseren et al., 2021). However, a cross-sectional study of 9,823 participants from 13 countries (CAPTURE) found that whilst one in three adults with T2D had cardiovascular disease, only one in five were receiving glucose-lowering agents with demonstrated cardiovascular benefit (Mosenzon et al., 2021).

The Middle East and North Africa have the highest percentage of diabetes-related deaths among people of working age (IDF, 2021). Globally, 14% of people with diabetes live in the Middle East and North Africa region, but only 3% of the global expenditure for diabetes care was spent there (IDF, 2021). The CAPTURE study had limited coverage of countries across the Middle East and Africa; thus, there is a need for updated evidence on the prevalence and management of ASCVD among adults with T2D in these two regions. To address this gap, we undertook a multicentre, cross-sectional chart review study to determine the prevalence and clinical management of ASCVD in patients with T2D in the Middle East and Africa (PACT-MEA) (Verma et al., 2023a). The PACT-MEA study was conducted in Bahrain, Egypt, Jordan, Kuwait, Qatar, South Africa, and the United Arab Emirates (UAE). The main findings from the chart audit revealed that one in five patients with T2D had established ASCVD and 99% met the 2021 European Society of Cardiology (ESC) high or very high risk criteria for ASCVD (Visseren et al., 2021; Verma et al., 2023b). However, no patients in the cohort achieved all ESC 2021 guideline targets for the prevention of cardiovascular disease (Visseren et al., 2021; Verma et al., 2023b). We aimed to assess the impact of clinical and non-clinical factors on treatment decision-making of physicians managing patients with T2D in primary and secondary care across the seven Middle Eastern and African countries.

## 2 Materials and methods

### 2.1 Study design and ethics

We conducted a cross-sectional study of physicians managing patients with T2D in the countries included in the PACT-MEA study between 13 June and 1 October, 2022. The design and rationale for the PACT-MEA study (NCT05317845), including this research has been published previously (Verma et al., 2023a).

This study was conducted in accordance with the Declaration of Helsinki and International Society for Pharmacoepidemiology (ISPE) guidelines for Good Clinical and Pharmacoepidemiology Practice (GPP). The study protocol and informed consent form were reviewed and approved by local Institutional Review Boards/Ethics Committees (IRB/EC) and other regulatory agencies as required for each participating country; a full list of IRB/EC institutions, approval dates, and reference numbers is provided at the end of the manuscript. The contract research organisation, IQVIA (Durham, NC, United States), was responsible for data management for the study. The physicians were informed of the study design and were asked to provide consent electronically before participating in the study. Adequate protections were taken to maintain the confidentiality of their responses. Data were managed in compliance with the General Data Protection Regulation and any regulations regarding management of personal data required by participants’ respective country of residence.

### 2.2 Setting

Physicians were recruited from the study site countries and completed the questionnaire online. Primary and secondary care facilities in Bahrain, Egypt, Jordan, Kuwait, Qatar, South Africa, and the UAE (Supplementary Appendix A) were selected as study sites for the chart review portion of the study based on local scientific or treatment guidelines for patients with T2D, reimbursement criteria, referral flows, and country-specific regulation governing site involvement in studies of this nature.

### 2.3 Study population

At least one investigator was anonymously recruited from each participating study site and additional physicians were recruited using IQVIA’s database of individuals who had previously opted in to participate in research but had not participated in the PACT-MEA study. Physicians were recruited from each of the seven participating countries, if they spent at least 50% of their time managing patients with T2D, had been in clinical practice for two or more years, and provided informed consent. The participating physicians were classified as primary care providers (PCPs) if they identified themselves as family practice physicians or general practitioners or as specialists if they identified themselves as endocrinologists, diabetologists, internal medicine physicians, or cardiologists. The diabetologists in South Africa are considered as primary care physicians due to their local healthcare system categorization, however for the purpose of this study, we have gathered inputs of the HCPs as per their relevant specialty, irrespective of the healthcare setting in which they are practicing.

It was determined that a sample size of at least 350 physicians would be sufficient to address the study objective based on 5% margin of error and a 95% confidence interval. The sample size for each country was calculated based on the total sample required for the study, proportionally adjusted for the country’s physician population and local dynamics.

Regarding sample representativeness, the study aimed to achieve it by selecting physicians as in the patient chart review with the aim of maximizing representativeness of the sample. Consequently, the number and types of physicians included from each country were chosen to reflect local healthcare dynamics, including population size and care settings.

### 2.4 Assessment instrument

The participating physicians were contacted by email and provided with a link to Decipher, the online platform. A questionnaire (Supplementary Appendix B) was used to evaluate physicians’ self-reported factors driving clinical decision-making (i.e., treatment, patient, practice, and physician factors; engagement in shared decision-making) for the management of T2D based on a series of statements using a five-point Likert scale from one (strongly agree) to five (strongly disagree). We report the proportion of physicians in agreement, defined as rating of “agree” or “strongly agree”. In one question, physicians were asked to rank factors they consider when selecting a glucose-lowering treatment for their patients with T2D. We report the proportion of physicians ranking their top three factors as well as the mean ranking of each factor among all physicians.

### 2.5 Statistical analysis

Descriptive statistical analysis of the data was conducted with Q Research Software (Displayr, Inc., Pyrmont, NSW, Australia) and Microsoft Excel (Microsoft 365, Redmond, WA, United States). The results are presented as mean (SD) or numbers and percentages. Statistical comparisons (t-tests for means and z-tests for proportions) were conducted with Q Research Software and the probability of type I error was set at p = 0.05.

## 3 Results

### 3.1 Study participant characteristics

A total of 385 physicians were included in the study; 63 were primary investigators involved in the primary PACT-MEA study and 322 were recruited specifically for this physician study. Among respondents, the most common physician specialty was general practitioner (n = 152, 39%). There were slightly more “specialist” respondents (endocrinologists, diabetologists, internal medicine physicians, and cardiologists; n = 203, 53%) than PCP respondents (family practice physicians and general practitioners; n = 182, 47%) (Table 1). The respondents estimated that they spent 84% of their time, on average, managing patients in clinical care settings (Table 1).

**TABLE 1:** Characteristics of the study participants.

	PCPs^a^ (n = 182)	Specialists^b^ (n = 203)	All physicians (n = 385)
Medical specialty, n (%)^c^			
Family practice physician	30 (16)	0 (0)	30 (8)
General practitioner	152 (84)	0 (0)	152 (39)
Endocrinologist	0 (0)	69 (34)	69 (18)
Diabetologist	0 (0)	28 (14)	28 (7)
Internal medicine physician	0 (0)	54 (27)	54 (14)
Cardiologist	0 (0)	52 (26)	52 (14)
Country, n (%)^c^			
Bahrain	22 (12)	12 (6)	34 (9)
Egypt	35 (19)	59 (29)	94 (24)
Jordan	28 (15)	30 (15)	58 (15)
Kuwait	20 (11)	16 (8)	36 (9)
Qatar	24 (13)	11 (5)	35 (9)
South Africa^d^	41 (23)	29 (14)	70 (18)
United Arab Emirates	12 (7)	46 (23)	58 (15)
Years in practice, mean (SD)	18 (11)	22 (10)	20 (11)
Proportion of time spent working, mean % (SD)			
Managing patients in clinical care	88 (12)	80 (17)	84 (15)
Research outside clinical care	8 (9)	12 (10)	10 (10)
Administrative tasks	10 (8)	12 (10)	11 (9)

^a^ PCPs included self-reported family practice physicians and general practitioners.

^b^ Specialists included self-reported endocrinologists, diabetologists, internal medicine physicians, and cardiologists.

^c^ Indicates numbers may not add to 100 due to rounding.

^d^ Diabetologists in South Africa are considered primary care physicians locally, but for this study, inputs were gathered based on HCPs, relevant specialties regardless of practice setting.

Abbreviations: PCP, primary care physician; SD, standard deviation.

### 3.2 Selection of glucose-lowering treatment

We asked the physicians to rank 12 factors (where one indicates most important and 12 indicates least important) they consider when selecting a glucose-lowering treatment for their adult patients with T2D. The factors which were most frequently ranked in the top three by the participants included efficacy (n = 280, 73%), cardiovascular safety (n = 198, 51%), cardiovascular benefit (n = 173, 45%), and hypoglycaemia (n = 165, 43%). The specialists had a higher mean ranking of ‘weight benefit’ than the PCPs when selecting glucose-lowering treatments (PCPs, 6.9; specialists, 6.0; p < 0.05). The PCPs had a higher mean ranking of ‘route of administration’ than the specialist physicians when selecting glucose-lowering treatments (PCPs, 7.3; specialists, 8.3; p < 0.05).

### 3.3 Impact of clinical factors on disease management decisions

When asked about clinical factors which impact their management decisions, physicians were most likely to agree that glycated hemoglobin (HbA1c) (n = 370, 96%), chronic kidney disease (n = 367, 95%), and high risk for ASCVD (n = 366, 95%) impact their management of patients with T2D. The physicians were least likely to agree that disease duration (n = 296, 77%) impacted their management decisions. The PCPs were less likely than the specialists to agree that disease duration influences their T2D management decisions (PCPs, n = 131, 72%; specialists, n = 165, 81%; p < 0.05).

### 3.4 Impact of patient-related factors on disease management decisions

**FIGURE 1:**
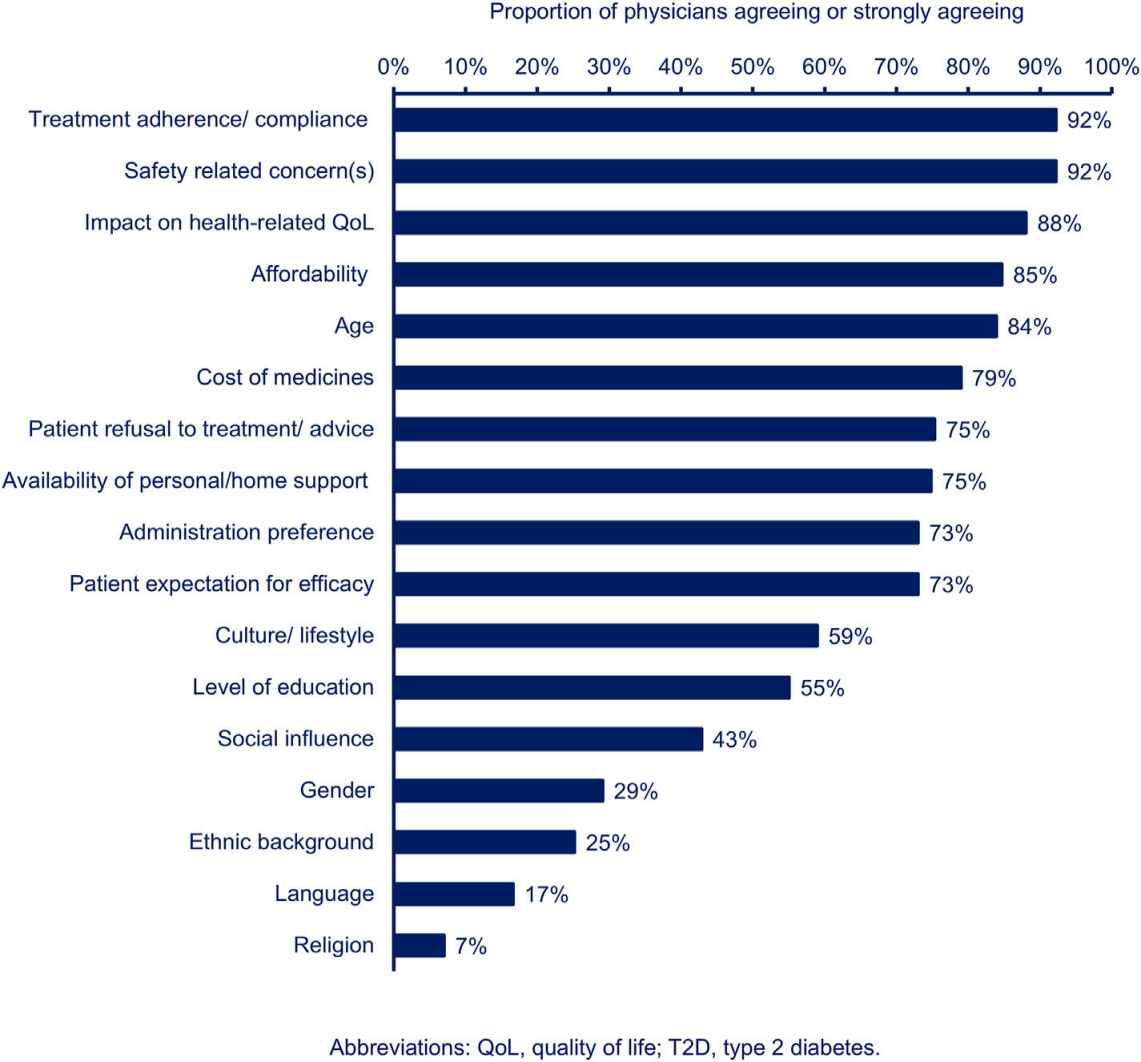
Proportion of physicians (n = 385) agreeing or strongly agreeing that each patient-related factor influences their T2D management decisions.

When asked about patient factors which influence their T2D management decisions, the physicians were most likely to agree that treatment adherence/compliance, safety concerns, impact on health-related quality of life (i.e., physical and psychosocial functional behaviour), affordability, and age influenced their decisions (Figure 1). The PCPs were more likely than the specialists to agree that patient age influences their T2D management decisions (PCPs, n = 161, 88%; specialists, n = 162, 80%; p < 0.05). The PCPs were less likely than the specialists to agree that cost of medicines influences their T2D management decisions (PCPs, n = 132, 73%; specialists, n = 172, 85%; p < 0.05).

### 3.5 Impact of practice-related factors on disease management decisions

The physicians were most likely to agree that availability of treatment (n = 335, 87%), insurance and reimbursement criteria (n = 279, 72%), and access to other healthcare professionals (n = 272, 71%) influenced their T2D management decisions in their practice setting. The PCPs were less likely than the specialists to agree that insurance and reimbursement criteria influenced their T2D management decisions (PCP, n = 120, 66%; specialist, n = 159, 78%; p < 0.05). The PCPs were more likely than the specialists to agree that access to other healthcare professionals influenced their T2D management decisions (PCP, n = 144, 79%; specialist, n = 128, 63%; p < 0.05).

### 3.6 Impact of physician-related factors on disease management decisions

**FIGURE 2:**
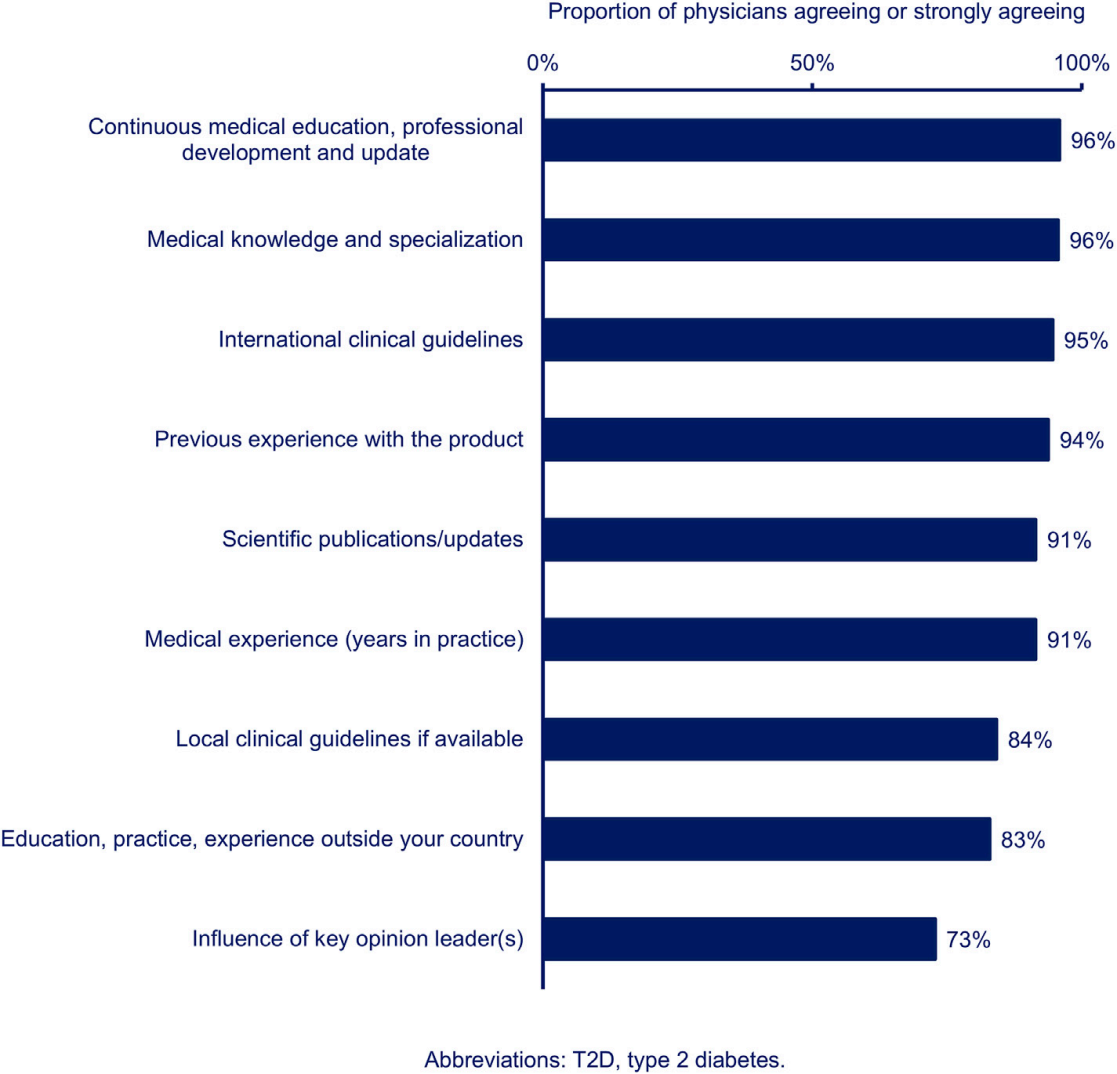
Proportion of physicians (n = 385) agreeing (combined agree and strongly agree) that each physician-related factor influences their T2D management decisions.

When asked about physician-related factors influencing their T2D management decisions, most physicians agreed with all listed items (Figure 2). The physicians were most likely to agree that continuous medical education, medical knowledge, international clinical guidelines, and previous experience with a product influenced their T2D management decisions (Figure 2).

### 3.7 Physician clinical decision-making approach

**FIGURE 3:**
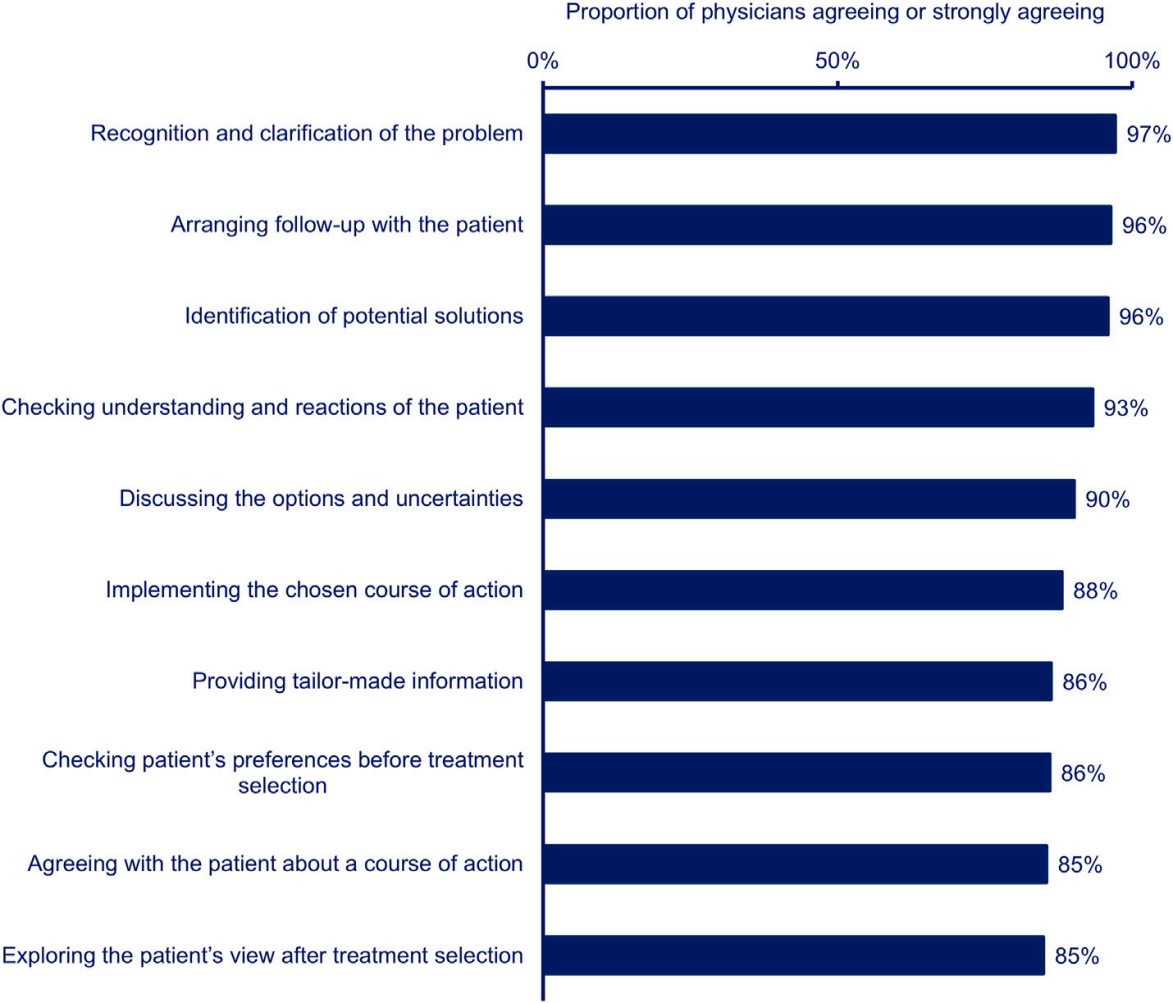
Proportion of physicians (n = 385) agreeing (combined agree and strongly agree) that each statement describes their clinical decision-making process.

When asked about their clinical decision-making process, physicians were most likely to agree that recognition and clarification of the problem, arranging follow-up with the patient, and identification of potential solutions described their clinical decision-making approach (Figure 3).

**FIGURE 4:**
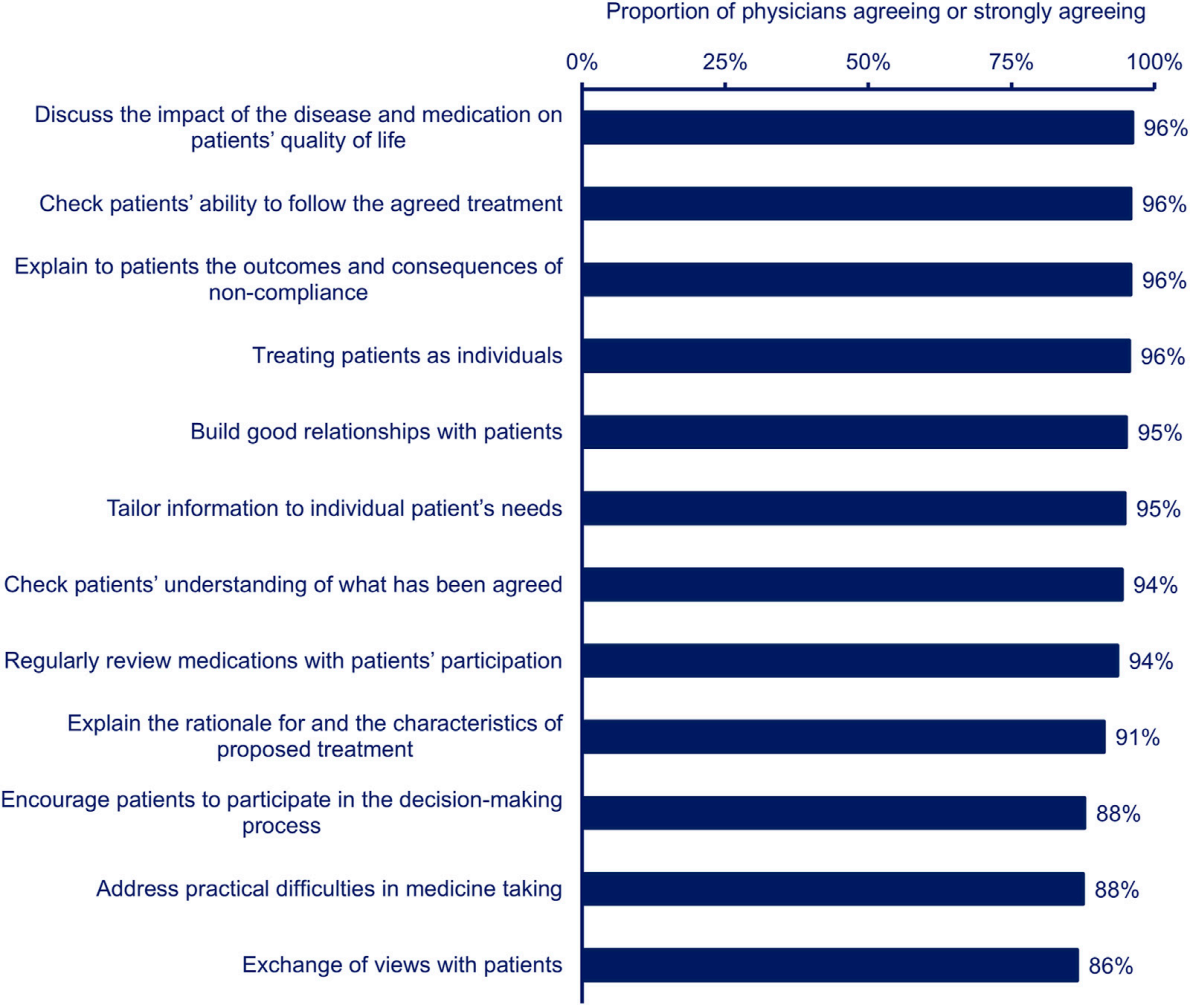
Proportion of physicians (n = 385) agreeing (combined agree and strongly agree) that each statement describes their clinical decision-making process in terms of patient empowerment.

Most physicians agreed with all statements regarding clinical decision-making in terms of patient empowerment (Figure 4). However, physicians were least likely to agree that encouraging patients to participate in the decision-making process, addressing practical difficulties in medicine taking, and exchange of views with patients influenced their decision-making process (Figure 4). The PCPs were more likely than the specialists to agree that exchanging views with patients influenced their clinical decision-making in terms of patient empowerment (PCP, n = 164, 90%; specialist, n = 169, 83%; p < 0.05).

### 3.8 Shared clinical decision-making

The physicians were asked whether they agreed with three potential strategies to improve shared decision-making in the management of patients with T2D. Most physicians agreed that improved communication skills of physicians/healthcare professionals (n = 372, 97%), coaching and question prompts for patients/effective dialogue (n = 351, 91%), and patient decision aids (i.e., providing information about options and outcomes) (n = 336, 87%) could improve shared decision-making. The PCPs were more likely than the specialists to agree that patient decision aids could improve shared decision-making (PCP, n = 169, 93%; specialist, n = 167, 82%; p < 0.05).

## 4 Discussion

This study highlights the importance of access to medications, non-clinical (patient and physician) factors, and continuing medical education in impacting decision-making related to the management of patients with T2D and high risk of ASCVD.

The chart audit component of the PACT-MEA study revealed that one in five patients with T2D had established ASCVD and 99% were at high or very high risk of ASCVD according to 2021 ESC guidelines (Visseren et al., 2021; Verma et al., 2023b). However, no patients met all ESC targets (HbA1c <7%, blood pressure <130/80 mm Hg, low-density lipoprotein cholesterol <1.8 mmol/L, use of SGLT2 inhibitors, use of GLP-1 RAs; exercising ≥5 times per week and maintaining body mass index <25 kg/m^2^) for the prevention of cardiovascular outcomes (Visseren et al., 2021; Verma et al., 2023b). Of the patients at high or very high risk of ASCVD, only 37% were treated with SGLT2 inhibitors and 13% were treated with GLP-1 RAs (Verma et al., 2023b). According to the American Diabetes Association (ADA) 2022 guidelines, there is evidence supporting significant cardiovascular benefits of four FDA-approved GLP-1 RAs (liraglutide, albiglutide, semaglutide, and dulaglutide) and three FDA-approved SGLT2 inhibitors (empagliflozin, canagliflozin, and dapagliflozin), with lesser benefits seen with ertugliflozin, for patients with T2D and high risk of ASCVD (AD A, 2022). Despite international consensus that patients with T2D can benefit from GLP-1 RAs and SGLT2 inhibitors and the fact that 95% of the physicians in this study agreed that international clinical guidelines strongly influence their T2D management decisions, only a minority of patients in the PACT-MEA chart audit were receiving these medications (Verma et al., 2023b). This finding aligns with global data; for example, studies from Denmark (2017) and Scotland (2019) studies have reported lower utilization of SGLT2is and GLP-1 RAs, despite their recommendations by local guidelines (Rungby et al., 2017; McGurnaghan et al., 2019). Similarly, in the US, during 2014–2015, data from a large cohort of 1,202,596 patients showed that less than 12% used either SGLT2is or GLP-1 RAs for T2D management (Weng et al., 2019). However, recent study indicates a shift in treatment patterns. A survey of 515 physicians across various specialties in the US reported much higher usage rates, 78% for SGLT2is and 67% for GLP-1 RAs (Yaseen and Lahiri, 2023) suggesting a growing trend toward increased adoption of these therapies.

Reimbursement of cardioprotective medications for patients with T2D varies across countries included in the PACT-MEA study (Supplementary Appendix C) and could partially explain prescribing patterns of such products in the two regions. Additionally, physicians in our study agreed that previous experience with a product influenced their T2D management decisions. Therefore, physicians may be less likely to prescribe novel medications with which they do not have prior experience. These discrepancies between self-reported practices and actual clinical implementation highlight regional variations. Supporting this, the ADA/EASD consensus emphasizes that access, treatment costs, and insurance coverage are critical factors influencing the selection of glucose-lowering medications. Cost and access to newer therapies remain significant barriers globally. The availability of glucose-lowering drugs, patient support systems, and blood glucose monitoring devices varies widely, influenced by regional economic, cultural, and healthcare factors. Within healthcare systems, medication coverage often depends on cost-effectiveness assessments (Davies et al., 2018).

Previous research on barriers to the delivery of diabetes care in the Middle East and South Africa conducted in 2013 found that physicians ranked patient lifestyles, lack of education and poor dietary compliance as the most important barriers to optimal diabetes control (Assaad-Khalil et al., 2013). However, physicians were less likely to rank access to medication, and poor medication compliance in their top three barriers to optimal diabetes control (Assaad-Khalil et al., 2013). Our study identified treatment adherence/compliance as the top patient-related factor, whilst 85% of the physicians agreed that affordability of medications influenced their T2D management decisions. These results may reflect the introduction of novel classes of medication and associated increasing costs of T2D pharmacotherapies between 2013 and 2023.

The participating physicians in our study highlighted the importance of patient- and physician-related factors when managing patients with T2D and the desire for continuing medical education. Three prominent factors included patient adherence to medications, access to other healthcare professionals, and improvement in communication skills of physicians. In other regions it has been shown that increases in patient activation (a patient’s willingness and ability to participate in care decisions) can reduce healthcare resource utilization (Begum et al., 2011) and, when coupled with functional health literacy, can improve glycaemic control (Woodard et al., 2014). Research on physician-patient shared decision-making in Saudi Arabia found that patients preferred a paternalistic approach (Alabdullah et al., 2023). In our study, physicians were more likely to agree that improving their communication skills would improve therapeutic outcomes, as opposed to coaching patients or utilizing patient decision aids–reflecting a paternalistic approach to healthcare. A study of patients with T2D in Saudi Arabia found that an intensive patient education programme led by a trained healthcare team resulted in improved diabetes control after 1 year (Neimat Mahmoud Abd-Alrahman Ali et al., 2019). Perhaps similar educational programmes can be utilized throughout the Middle East and Africa, in addition to continuous medical education for physicians, to improve patient activation and T2D outcomes in the two regions. Our study also indicated that most physicians believe improving communication, coaching, question prompts, and patient decision aids can enhance shared decision-making. This reflects a positive attitude towards active patient involvement in treatment decision-making. Promoting shared decision-making supports patient autonomy, values, and commitment, thereby improving continuity of care (Say et al., 2006).

### 4.1 Policy implications

This study provides real-world evidence that can inform several policy initiatives aimed at improving diabetes care. Key areas of focus include enhancing access to specialized diabetic and cardiovascular services, as well as improving patients’ knowledge about T2D management. Such improvements are essential to promote treatment adherence and encourage healthier lifestyle changes.

#### 4.1.1 Strengthen access to and availability of treatments

Policymakers should ensure the consistent availability and affordability of medications and treatment options across healthcare facilities. This requires collaboration among stakeholders responsible for diabetic and cardiovascular care, including the Ministry of Health, reimbursement agencies, payers, patient organizations, and charitable groups.

#### 4.1.2 Enhance patient engagement and education

Investing in the development and funding of patient education tools and programmes to improve patient understanding of T2D management, medication adherence, and lifestyle modifications.

#### 4.1.3 Improve communication skills and training for HCPs

Integrating communication skills training into medical education curriculum can strengthen doctor-patient relationships. Improved communication fosters shared decision-making, leading to better treatment adherence and outcomes.

#### 4.1.4 Foster strategic partnerships

Strengthening collaboration between healthcare authorities and clinicians is vital. Such partnerships can support the development, implementation, and evaluation of policies and programs that address existing barriers. This approach can also enable general physicians to prescribe SGLT2 inhibitors or GLP-1 RAs within primary healthcare settings, ensuring broader access to these effective therapies.

### 4.2 Strengths and limitations

Combining the outcomes of this physician study with that of the PACT-MEA chart audit allows us to contextualize physician attitudes and behaviours alongside regional ASCVD risk/prevalence and medication prescribing patterns. This has provided important insights into the complex basis of poor T2D control and poor uptake of cardioprotective therapies. The fact that our study participants spent more than half of their practice time managing patients with T2D adds strength to the findings of this study.

As a self-reported cross-sectional survey, this study has potential limitations, including social desirability and recall biases, which are common in survey-based research. Social desirability bias may lead physicians to overreport positive behaviors and underreport negative ones. To mitigate this, anonymization and cross-validation through chart reviews were implemented. Anonymization encourages more honest responses, while cross-validation helps assess data reliability and validity by comparing self-reports against different methods.

The study relied on self-reported data which is an excellent way to understand attitudes and behaviours surrounding the respective healthcare environments in a real world setting but can be prone to bias and/or inaccurate recall. Although some responses may overstate certain physician attitudes and behaviours, the aggregate responses to a given question can highlight the perceived relative importance of various factors impacting clinical decisions. The differences in characteristics (such as age, years of practice) between non-participating doctors and participants were not assessed, which may affect the generalizability of the results. Further, this study did not include as an objective quantification of health insurance coverage rate and out-of-pocket ratio in each of the participating countries as well as their reimbursement policies for the two types of drugs. Therefore, it was not possible to examine the relationship between such policies and the prescription rate for the two drugs.

While the study sites were selected with the aim of achieving a sample representative of the countries included, there could be significant differences between responders and non-responders. The sample sizes for some countries were relatively small and therefore limited our ability to compare responses between countries. As such, we focused on the aggregate data while highlighting some key differences between responses from different physician specialties. We broadly grouped physicians as PCPs or specialists based on their self-reported classifications. However, healthcare systems are organised differently in the countries included in this study. Physicians specializing in internal medicine are classified as primary care in some countries but as secondary care in others.

There is remote possibility for bias in reporting studies due to pharmaceutical industry sponsorship. It is perceived that the industry-sponsored research may be more likely to report positive results for the sponsored drug compared to independently funded studies, from study design to selective reporting of outcomes. However, this is not a perspective shared by the wider research community. As for this study, we did not compare a sponsored drug, and the investigators were closely engaged in the conceptualization, design, execution, analysis, interpretation, and reporting of the study. Additionally, an independent Study Steering Committee provided comprehensive oversight from start to finish.

### 4.3 Conclusion

In conclusion, the physicians in this study identified the importance of medication access/availability and non-clinical (patient and physician) factors in influencing their management of patients with T2D. We have identified an opportunity to improve the patient-doctor relationship and shared decision-making to optimise the use of therapies that enhance patient outcomes.

## 5 Plain language summary

The Middle East and Africa have some of the highest rates of type 2 diabetes (T2D) and diabetes-related deaths in the world. Effectively managing diabetes can help improve the health of patients with the disease in these regions. We explored how doctors in the Middle East and Africa make treatment decisions for their patients with T2D. What factors affect their decision-making? How could decision-making be improved? We gathered the views of 385 doctors in seven countries (Bahrain, Egypt, Jordan, Kuwait, Qatar, South Africa, and the UAE) in 2022. Doctors were asked to rate one (strongly agree) to five (strongly disagree) from a list of patient factors, office setting factors, and personal factors that might influence their decision-making. The patient factors which most influenced their T2D management decisions were patients taking their medication as prescribed (92%), safety concerns (92%), and impact on health-related quality of life (88%). The availability of treatment (87%) was the most influential office setting factor. The three personal factors which most influenced their decision-making included continuous medical education (96%), medical knowledge (96%), and international medical standards (95%). Most doctors agreed that improved communication skills of doctors (97%), training and question prompts for patients (91%), and patient education tools (87%) could improve the patient-doctor relationship. This could help patients and doctors work together to make decisions about patients’ healthcare.

## Data Availability

The original contributions presented in the study are included in the article/Supplementary Material, further inquiries can be directed to the corresponding author.

## References

[B1] ADA (2022). 10. Cardiovascular disease and risk management: standards of medical care in Diabetes-2022. Diabetes Care 45, S144–S174. 10.2337/dc22-S010 34964815

[B2] ADA, 9 (2021). 9. Pharmacologic approaches to glycemic treatment: standards of medical care in Diabetes-2022. Diabetes Care 45, S125–S143. 10.2337/dc22-S009 34964831

[B3] Alabdullah Y. Y. Alzaid E. Alsaad S. Alamri T. Alolayan S. W. Bah S. (2023). Autonomy and paternalism in shared decision-making in a Saudi Arabian tertiary hospital: a cross-sectional study. Dev. World Bioeth. 23, 260–268. 10.1111/dewb.12355 35586963

[B4] Assaad-Khalil S. H. Al Arouj M. Almaatouq M. Amod A. Assaad S, N. Azar S. T. (2013). Barriers to the delivery of diabetes care in the Middle East and South Africa: a survey of 1,082 practising physicians in five countries. Int. J. Clin. Pract. 67, 1144–1150. 10.1111/ijcp.12205 24165428

[B5] Begum N. Donald M. Ozolins I. Z. Dower J. (2011). Hospital admissions, emergency department utilisation and patient activation for self-management among people with diabetes. Diabetes Res. Clin. Pract. 93, 260–267. 10.1016/j.diabres.2011.05.031 21684030

[B6] Davies M. J. D’Alessio D. A. Fradkin J. Kernan W. N. Mathieu C. Mingrone G. (2018). Management of hyperglycemia in type 2 diabetes, 2018. A consensus report by the American diabetes Association (ADA) and the european association for the study of Diabetes (EASD). Diabetes care 41 (12), 2669–2701. 10.2337/dci18-0033 30291106 PMC6245208

[B7] Gerstein H. C. (2015). Diabetes: dysglycaemia as a cause of cardiovascular outcomes. Nat. Rev. Endocrinol. 11, 508–510. 10.1038/nrendo.2015.118 26215261

[B8] Hudspeth B. (2018). The burden of cardiovascular disease in patients with diabetes. Am. J. Manag. Care 24, S268-S272–s272. 30160393

[B9] IDF (2021). “IDF diabetes atlas,” in Brussels, Belgium.

[B10] Marx N. Federici M. Schütt K. Müller-Wieland D. Ajjan R. A. Antunes M. J. (2023). ESC guidelines for the management of cardiovascular disease in patients with diabetes: developed by the task force on the management of cardiovascular disease in patients with diabetes of the european society of cardiology (ESC). Eur. Heart J., ehad192. 10.1093/eurheartj/ehad192 38195096

[B11] McGurnaghan S. Blackbourn L. A. Mocevic E. Haagen Panton U. McCrimmon R. J. Sattar N. (2019). Cardiovascular disease prevalence and risk factor prevalence in type 2 diabetes: a contemporary analysis. Diabetic Med. 36 (6), 718–725. 10.1111/dme.13825 30246473 PMC6585697

[B12] Mosenzon O. Alguwaihes A. Leon J. L. A. Bayram F. Darmon P. Davis T. M. E. (2021). CAPTURE: a multinational, cross-sectional study of cardiovascular disease prevalence in adults with type 2 diabetes across 13 countries. Cardiovasc. Diabetol. 20, 154. 10.1186/s12933-021-01344-0 34315481 PMC8317423

[B13] Neimat Mahmoud Abd-Alrahman Ali D. Ghassan Abd-Al lateef Mohammad Al S. Mohamed Abdalla E. Ahmed Fadlala Ahmed A. Jamaan Ahmed Alghamdi H. Ali Alghamdi A. (2019). Effect of diabetes educational program on self-care and diabetes control among type 2 diabetic patients in Al-Baha–Saudi arabia. AIMS Med. Sci. 6, 239–249. 10.3934/medsci.2019.3.239

[B14] Ong K. L. Stafford L. K. McLaughlin S. A. Boyko E. J. Emil Vollset S. Smith A. E. (2023). Global, regional, and national burden of diabetes from 1990 to 2021, with projections of prevalence to 2050: a systematic analysis for the global burden of disease study 2021. Lancet 402, 203–234. 10.1016/S0140-6736(23)01301-6 37356446 PMC10364581

[B15] Rungby J. Schou M. Warrer P. Ytte L. Andersen G. S. (2017). Prevalence of cardiovascular disease and evaluation of standard of care in type 2 diabetes: a nationwide study in primary care. Cardiovasc. Endocrinol. and Metabolism 6 (4), 145–151. 10.1097/XCE.0000000000000135 29276653 PMC5704655

[B16] Say R. Murtagh M. Thomson R. (2006). Patients’ preference for involvement in medical decision making: a narrative review. Patient Educ. Couns. 60 (2), 102–114. 10.1016/j.pec.2005.02.003 16442453

[B17] Verma S. Sabbour H. Alamuddin N. Alawadi F. Alkandari H. Almahmeed W. (2023a). A cross-sectional study of the prevalence and clinical management of atherosclerotic cardiovascular diseases in patients with type 2 diabetes across the Middle East and Africa (PACT-MEA): study design and rationale. Obes. Metabolism 25, 1444–1452. 10.1111/dom.15011 36775980

[B18] Verma S. Alamuddin N. Alawadi F. Alkandari H. Almahmeed W. Assaad-Khalil S. H. (2023b). Prevalence of diabetes and cardiovascular risk in the Middle east and africa: primary results of the PACT-MEA study. Circulation 147, 1251–1255. 10.1161/CIRCULATIONAHA.123.064345 36877670 PMC10101130

[B19] Visseren F. L. J. Mach F. Smulders Y. M. Carballo D. Koskinas K. C. Bäck M. (2021). ESC guidelines on cardiovascular disease prevention in clinical practice: developed by the task force for cardiovascular disease prevention in clinical practice with representatives of the european society of cardiology and 12 medical societies with the special contribution of the European Association of preventive cardiology (EAPC). Eur. Heart J. 42, 3227–3337. 10.1093/eurheartj/ehab484 34458905

[B20] Weng W. Tian Y. Kong S. X. Ganguly R. Hersloev M. Brett J. (2019). The prevalence of cardiovascular disease and antidiabetes treatment characteristics among a large type 2 diabetes population in the United States. Endocrinol. Diabetes and Metabolism 2 (3), e00076. 10.1002/edm2.76 31294089 PMC6613222

[B21] Woodard L. D. Landrum C. R. Amspoker A. B. Ramsey D. Naik A. D. (2014). Interaction between functional health literacy, patient activation, and glycemic control. Patient Prefer Adherence 8, 1019–1024. 10.2147/PPA.S63954 25092966 PMC4114908

[B22] Yaseen A. Lahiri S. W. (2023). Health care provider prescribing habits and barriers to use of new type 2 diabetes medications: a single-system survey study. Clin. Diabetes 41 (4), 490–501. 10.2337/cd22-0100 37849520 PMC10577502

