# Supplementary material for: Physician self-reported factors driving clinical decision-making in management of patients with T2D and ASCVD/high risk of ASCVD across the middle East and Africa: a cross-sectional study
Source: Front Pharmacol. 2025 Sep 9;16:1558515. doi: 10.3389/fphar.2025.1558515 (PMC12455046; doi:10.3389/fphar.2025.1558515)
Supplement: Supplementary file 1 [file DataSheet2.pdf]

STROBE Statement—checklist of items that should be included in reports of observational studies

	Item No.	Recommendation	Page No.	Relevant text from manuscript
**Title and abstract**	1	(*a*) Indicate the study's design with a commonly used term in the title or the abstract	1	a cross-sectional study
		(*b*) Provide in the abstract an informative and balanced summary of what was done and what was found	7	(see abstract)
**Introduction**				
Background/rationale	2	Explain the scientific background and rationale for the investigation being reported	9	(see introduction)
Objectives	3	State specific objectives, including any prespecified hypotheses	10	"we aimed to assess the impact of clinical and non-clinical factors on treatment decision-making of physicians managing patients with T2D in primary and secondary care across the 7 Middle Eastern and African countries."
**Methods**				
Study design	4	Present key elements of study design early in the paper	10	(see 'study design and ethics' section)
Setting	5	Describe the setting, locations, and relevant dates, including periods of recruitment, exposure, follow-up, and data collection	10-11	(see 'study design and ethics' section and 'setting' section)
Participants	6	(*a*) *Cohort study*—Give the eligibility criteria, and the sources and methods of selection of participants. Describe methods of follow-up<br>*Case-control study*—Give the eligibility criteria, and the sources and methods of case ascertainment and control selection. Give the rationale for the choice of cases and controls<br>*Cross-sectional study*—Give the eligibility criteria, and the sources and methods of selection of participants	11-12	(see 'study population' section)
		(*b*) *Cohort study*—For matched studies, give matching criteria and number of exposed and unexposed<br>*Case-control study*—For matched studies, give matching criteria and the number of controls per	NA	NA

		case		
Variables	7	Clearly define all outcomes, exposures, predictors, potential confounders, and effect modifiers. Give diagnostic criteria, if applicable	12	(see ‘Assessment instrument’ section)
Data sources/ measurement	8*	For each variable of interest, give sources of data and details of methods of assessment (measurement). Describe comparability of assessment methods if there is more than one group	12	(see ‘Assessment instrument’ section)
Bias	9	Describe any efforts to address potential sources of bias	23	(see ‘Strengths and Limitations’ section in Discussion)
Study size	10	Explain how the study size was arrived at	12	“It was determined that a sample size of at least 350 physicians would be sufficient to address the study objective based on 5% margin of error and 95% confidence interval. The overall sample size was distributed across each participating country according to their physician population.”

Quantitative variables	11	Explain how quantitative variables were handled in the analyses. If applicable, describe which groupings were chosen and why	12-13	“The participating physicians were classified as primary care providers (PCPs) if they identified themselves as family practice physicians or general practitioners or as specialists if they identified themselves as endocrinologists, diabetologists, internal medicine physicians, or cardiologists. Although diabetologists are classified under primary care in South Africa, we classified South African diabetologists as specialists.”<br>“A questionnaire (Supplementary material S2) was used to evaluate physicians’ self-reported factors driving clinical decision-making (i.e., treatment, patient, practice, and physician factors; engagement in shared decision-making) for the management of T2D based on a series of statements using a five-point Likert scale from one (strongly agree) to five (strongly disagree). We report the proportion of physicians in agreement, defined as rating of ‘agree’ or ‘strongly agree’. In one question, physicians were asked to rank factors they consider when selecting a glucose-lowering treatment for their patients

				with T2D. We report the proportion of physicians ranking their top 3 factors as well as the mean ranking of each factor among all physicians.”
Statistical methods	12	(*a*) Describe all statistical methods, including those used to control for confounding	12-13	(see ‘Statistical analysis’ section)
		(*b*) Describe any methods used to examine subgroups and interactions	12-13	(see ‘Statistical analysis’ section)
		(*c*) Explain how missing data were addressed	NA	NA
		(*d*) *Cohort study*—If applicable, explain how loss to follow-up was addressed<br>*Case-control study*—If applicable, explain how matching of cases and controls was addressed<br>*Cross-sectional study*—If applicable, describe analytical methods taking account of sampling strategy	11-12	“It was determined that a sample size of at least 350 physicians would be sufficient to address the study objective based on 5% margin of error and 95% confidence interval. The overall sample size was distributed across each participating country according to their physician population.”
		(*e*) Describe any sensitivity analyses	NA	NA
**Results**				
Participants	13*	(a) Report numbers of individuals at each stage of study—eg numbers potentially eligible, examined for eligibility, confirmed eligible, included in the study, completing follow-up, and analysed	13-14	“A total of 385 physicians were included in the study; 63 were primary investigators involved in the primary PACT-MEA study and 322 were recruited specifically for the physician survey. Among respondents, the most common physician specialty was general practitioner (n = 152, 39%). There were slightly more ‘specialist’ respondents (endocrinologists,

				diabetologists, internal medicine physicians, and cardiologists; n = 203, 53%) than PCP respondents (family practice physicians and general practitioners; n = 182, 47%)”
		(b) Give reasons for non-participation at each stage	NA	NA
		(c) Consider use of a flow diagram	NA	NA
Descriptive data	14*	(a) Give characteristics of study participants (eg demographic, clinical, social) and information on exposures and potential confounders	13-14	(See Table 1. Characteristics of study participants)
		(b) Indicate number of participants with missing data for each variable of interest	NA	NA
		(c) *Cohort study*—Summarise follow-up time (eg, average and total amount)	NA	NA
Outcome data	15*	*Cohort study*—Report numbers of outcome events or summary measures over time	*NA*	*NA*
		Case-control study—Report numbers in each exposure category, or summary measures of exposure	*NA*	*NA*
		Cross-sectional study—Report numbers of outcome events or summary measures	*14-19*	(see Results section)
Main results	16	(*a*) Give unadjusted estimates and, if applicable, confounder-adjusted estimates and their precision (eg, 95% confidence interval). Make clear which confounders were adjusted for and why they were included	NA	NA
		(*b*) Report category boundaries when continuous variables were categorized	12	“We report the proportion of physicians in agreement, defined as rating of ‘agree’ or ‘strongly agree’. In one question, physicians were asked to rank factors they consider when selecting a glucose-lowering treatment for their patients with T2D. We report the proportion of physicians ranking their top 3 factors as well as the mean ranking of each factor among all physicians.”
		(*c*) If relevant, consider translating estimates of relative risk into absolute risk for a meaningful time	NA	NA

		period		
Other analyses	17	Report other analyses done—eg analyses of subgroups and interactions, and sensitivity analyses	NA	NA
**Discussion**				
Key results	18	Summarise key results with reference to study objectives	21	“This study highlights the importance of access to medications, non-clinical (patient and physician) factors, and continuing medical education in impacting decision-making related to the management of patients with T2D and high risk of ASCVD.”
Limitations	19	Discuss limitations of the study, taking into account sources of potential bias or imprecision. Discuss both direction and magnitude of any potential bias	23	(see ‘Strengths and limitations’ section)
Interpretation	20	Give a cautious overall interpretation of results considering objectives, limitations, multiplicity of analyses, results from similar studies, and other relevant evidence	21-24	(see Discussion)
Generalisability	21	Discuss the generalisability (external validity) of the study results	23	(see ‘Strengths and limitations’ section)
**Other information**				
Funding	22	Give the source of funding and the role of the funders for the present study and, if applicable, for the original study on which the present article is based	25	(see ‘Funding’ section)

*Give information separately for cases and controls in case-control studies and, if applicable, for exposed and unexposed groups in cohort and cross-sectional studies.

Note: An Explanation and Elaboration article discusses each checklist item and gives methodological background and published examples of transparent reporting. The STROBE checklist is best used in conjunction with this article (freely available on the Web sites of PLoS Medicine at http://www.plosmedicine.org/, Annals of Internal Medicine at http://www.annals.org/, and Epidemiology at http://www.epidem.com/). Information on the STROBE Initiative is available at www.strobe-statement.org.
