# Supplementary material for: Physician self-reported factors driving clinical decision-making in management of patients with T2D and ASCVD/high risk of ASCVD across the middle East and Africa: a cross-sectional study
Source: Front Pharmacol. 2025 Sep 9;16:1558515. doi: 10.3389/fphar.2025.1558515 (PMC12455046; doi:10.3389/fphar.2025.1558515)
Supplement: Supplementary file 2 [file DataSheet1.pdf]

Supplementary appendices

Appendix A. Study site list.

Country	Principial Investigator	Site Name
Bahrain	Houssam Nour	Awali Cardiac Center
Bahrain	Saeed Khalaf	Salmaniya Medical Center
Bahrain	Jehan Abdulla	Bahrain Defence Force Hospital
Bahrain	Naji Alamuddin	King Hamad University Hospital
Egypt	Salwa Seddik	Ain Shams University
Egypt	Samir Helmy Assaad Khalil	Alexandria University
Egypt	Nabil El Kafrawy	Menoufia university
Egypt	Manal Abu Shady	Ain Shams University
Egypt	Hesham El Hefnawy	NIDE
Egypt	Atef Bassuoiny	NIDE
Egypt	Lobna El Touni	Assuit University
Egypt	Magdy Helmy	Alexandria University
Egypt	Hanan Gawish	Mansoura University
Egypt	Ibrahim El Ebrashi	Cairo University
Jordan	Jihad Haddad	Private Clinic
Jordan	Nidal Khatib	Ibn Al Haitham Hospital

Jordan	Firas Abbass	Islamic Hospital
Jordan	Fares Haddad	Abdali Medical Center
Jordan	Mustafa Jaradat	Jabal Al Zaitoun Hospital
Jordan	Munir Abu Al Samen	Private Clinic
Jordan	Hiba Abassi	Jordan University Hospital
Jordan	Eyas Al Mousa	Istishari Hospital
Jordan	Fawaz Ammari	King Abdullah University Hospital
Jordan	Adi Khasawneh	JUST Center
Kuwait	Hessa Alkandari	Farwaniya Hospital
Kuwait	Doaa Khalifa Hussein	Yarmuk clinic
Kuwait	Abeer AlFadel	Omarya Health Center
Kuwait	Sara Alwazaq	Jahra Hospital
Kuwait	Hanan Al-Fedhala	West Mishrif Health Center
Kuwait	Waleed AlDahi	Mubarak alKabeer Hospital
Qatar	Rayaz A Malik	Weill Cornell Medicine-Qatar
Qatar	Amin Jayoussi	Hamad General Hospital
Qatar	Mohammed Ali	Al Daayen Health Centre
Qatar	Tarik El Hadd	Al Wakrah Hospital
Qatar	Tahir Shaheen	South Al Wakra health centre

Qatar	Sohail Ahmed	Um Salal Health Centre
Qatar	Nazia Edathola Kottasseri	Qatar University Health Care
South Africa	Sindeep Bhana	Scott Street, Waverley
South Africa	Zaheer Bayat	Sandton Mediclinic
South Africa	Tracy-Lee Janse van Rensburg	Claremont
South Africa	Landman Lombard	Cape Town Medical Research Centre
South Africa	Ntobeko Ntusi	Groote Schuur Hospital Cardiology Dept.
South Africa	Hemant Makan	Lenasia
South Africa	Naeem Moosa	Shop 6 Freeway Plaza, Lenasia
South Africa	Shaifali Joshi	Brooklyn
South Africa	Sundeep Ruder	Life Hospital, Fourways
South Africa	Mukesh Joshi	Diabetes Care Centre & CDE Centre
South Africa	Gracjan Podgorski	Netcare Greenacres Hospital
South Africa	Alkesh Magan	Sandton Mediclinic
South Africa	Adri Kok	Netcare Union Hospital
South Africa	L Mayet	CDE, Houghton Group Practice

South Africa	Hilton Kaplan	Dr Hilton Kaplan Inc.
South Africa	Julien Trokis	Langeberg Medical Centre
UAE	Khadija Hafidh	Rashid Hospital
UAE	Fatheya Al Awadi	Dubai Hospital
UAE	Maher Jallo	Thumbay University Hospital
UAE	Zeenat Naseeb	Al Qassimi Hospital
UAE	Jalal Nafach	Dubai Diabetes Center
UAE	Vani Krishna Warier	Aster Clinics
UAE	Akrem El Malti	Rashid Center for Diabetes & Research
UAE	Hussien Heshmat	Fujairah Hospital
UAE	Hani Sabbour	Cleveland Clinic
UAE	Dinesh Dhanwal	NMC Abu Dhabi
UAE	Nader Lessan	ICLDC

Abbreviations: CDE, Cardiovascular Diabetes Education; ICLDC, Imperial College London Diabetes Centre; JUST, Jordan University of Science and Technology; NMC, New Medical Centre; UAE, United Arab Emirates.

Appendix B. Questionnaire.

<u>Inclusion Criteria</u>

- Signed informed consent before completion of the survey.
- Is the physician managing T2D patients.
- Practices in one of the participating countries: Bahrain, Kuwait, Qatar, UAE, Egypt, Jordan, South Africa.
- In clinical practice ≥ 2 years.
- Spends ≥ 50% of time managing patients in clinical care.

Specialty Quota:

- Primary Care: Family Medicine Physician, General Practitioner
- Secondary Care: Endocrinologist, Diabetologist, Internal Medicine, Cardiologist

SCREENER

PARTICIPANT INFORMATION SHEET

Dear Participant,

You *are being invited to participate in this survey which is part of PACT-MEA Disease Area Study (A multicenter, cross-sectional chart review and survey to capture the* ***P****revalence and clinical management of* ***A****therosclerotic* ***C****ardiovascular diseases in patients with* ***T****ype 2 diabetes across countries in the* ***M****iddle* ***E****ast and* ***A****frica (PACT-MEA))*

Thank you for taking the time to participate in this important research. IQVIA is clinical research organization requesting your consent to participate in this research survey and is the data controller. For your convenience we are offering this survey in multiple languages. Please select a language that you are most familiar with.

1. English
2. Arabic

Purpose of the study

The aim of the study is to identify the physician self-reported drivers in clinical decision-making in the management of type 2 diabetes (T2D) with established atherosclerotic cardiovascular diseases (ASCVD) or high ASCVD risk. This survey is designed to be complementary to the above stated PACT-MEA Disease Area Study sponsored by Novo Nordisk, a Danish research based pharmaceutical company.

Why you were selected?

You were selected as a possible participant from 350 other participating physicians because you are a physician managing T2D patients in one of the participating countries: Bahrain, Kuwait, Qatar, UAE, Egypt, Jordan, South Africa.

Information for participants

Participating in this questionnaire is **voluntarily**. There are no known disadvantages or costs whether you participate or not in this study. You may not benefit directly from this study but the information obtained will make a scientific contribution to the clinical decision-making process in the management of T2D through a publication in a peer-reviewed scientific journal once the study is completed. If you qualify and complete this survey, you will receive online panel credit listed in your invitation. The reimbursement will be done through IQVIA Mebos (IQVIA visa card) which can be used as a physical card or online as per our approved FMV for each country per specialty. The reimbursement will be done as per NN fair market value.

Confidentiality

You and your answers will not be identified, and no one will know whether or not you participated in the survey study (double blind). Your name will never be included in the report, publication, or identified to the sponsor. No individual information will be disclosed and the information you provide will be treated and kept strictly confidential. The study protocol will be reviewed by 'The Research Ethics Committee/Institutional Review Board' and 'National regulatory health authorities' with the aim to ensure appropriate regulatory and ethical approval of the study.

This research is being overseen by the Institutional Review Board (IRB),

……………………………… who perform independent review of research studies. You may contact the IRB at contact number ……………………. or E-mail if

you have questions, concerns, or complaints that are not being answered by the research team or have questions about your rights as a research subject.

Archiving and Documentation

All information from this study will be archived for at least 50 years after the Study Report is completed, or according to local requirements if longer archival duration is required. Your responses will be stored in secure servers in Ireland where IQVIA's data server is located. A final report of the survey study can be mailed to you, should you require it.

What do you need to do?

If you choose to participate in this survey, you are kindly asked to complete an online 20-minute survey. You are free to withdraw from the survey study or not to answer any particular question for any reason. This survey is anonymous so please do not write your name on the questionnaire. Please make sure to read carefully and acknowledge the informed consent part before you start completing the survey. A permanent record of your consent will be maintained.

You may contact DMBangalorePOC@iqvia.com with any questions, concerns or if you need additional support.

A description of this study will be available on http://www.clinicaltrials.gov. This website will not include information that can identify you. At most, the website will include a summary of the results. You can search this website at any time.

INFORMED CONSENT

S1 Do you consent to these terms and wish to continue with the survey?

1. Yes [CONTINUE]
2. No [NOT QUALIFIED]

[IF AGREES TO DISCLOSURE STATEMENT (S1/1), CONTINUE S2. ALL OTHERS TERMINATE.]

S2 Please note that this is a non-interventional study not related to any pharmaceutical or medical device product. Throughout the survey study if you raise an adverse event or a quality complaint in relation to a pharmaceutical product, this may need to be reported as per the pharmacovigilance requirements.

In such a situation, you will be contacted to ask whether you are willing to waive your confidentiality specifically related to the adverse event or quality complaint. All other information you provide will remain confidential. If we contact you, you may choose to accept or deny waiving confidentiality. It will not affect your participation in this study.

Do you understand this obligation to collect and report adverse events and quality complaints?

1. Yes
2. No

[IF UNDERSTANDS AE STATEMENT (S2/1) ASK S3, OTHERS TERMINATE]

ALL RESPONDENTS WHO CONSENT AND UNDERSTANDS AE STATEMENT (S1r1 and S2r1)

S3 What is your medical specialty?

1. Endocrinologist
2. Diabetologist

3. Family Practice Physician
4. General Practitioner
5. Internal Medicine Physician
6. Cardiologist

S4 What percentage of your professional time is spent performing each of the following activities?

Your best estimate will do. Your responses must sum to 100

[SHOW TOTAL SUM INDICATOR; MUST TOTAL TO 100] [RANGE 0-100]

1. Managing patients in clinical care I_I_I_I%
2. Research outside clinical care I_I_I_I%
3. Administrative tasks I_I_I_I%

[≥50% TIME IN MANAGING PATIENTS IN CLINICAL CARE (S4_1 >49) CONTINUE, OTHERWISE TERMINATE]

S5 In what [insert custom wording for each country] is the practice where you spend most of your time located

[INSERT DROP DOWN MENU]

1. Bahrain
2. Egypt
3. Jordan

4. Kuwait
5. Qatar
6. South Africa
7. UAE
8. None of the above [Terminate]

For **Bahrain** Display

In what region of Bahrain is the practice where you spend most of your time located?

[INSERT DROP DOWN MENU]

1. The Capital
2. Northern
3. Southern
4. Muharraq

For **Egypt** Display

In what region of Egypt is the practice where you spend most of your time located?

[INSERT DROP DOWN MENU]

1. Alexandria Governorate
2. Aswan Governorate
3. Asyut Governorate
4. Beheira Governorate

5. Beni Suef Governorate
6. Cairo Governorate
7. Dakahlia Governorate
8. Damietta Governorate
9. Faiyum Governorate
10. Gharbia Governorate
11. Giza Governorate
12. Ismailia Governorate
13. Kafr El Sheikh Governorate
14. Luxor Governorate
15. Matruh Governorate
16. Minya Governorate
17. Monufia Governorate
18. New Valley Governorate
19. North Sinai Governorate
20. Port Said Governorate
21. Qalyubia Governorate
22. Qena Governorate

23. Red Sea Governorate

24. Sharqia Governorate

25. Sohag Governorate

26. South Sinai Governorate

27. Suez Governorate

For **Jordan** Display

In what region of Jordan is the practice where you spend most of your time located?

[INSERT DROP DOWN MENU]

1. Ajloun
2. Amman
3. Aqaba
4. Balqa
5. Irdib
6. Jerash
7. Karak
8. Maan
9. Madaba
10. Mafraq

11. Tafileh
12. Zarqa

For **Kuwait** Display

In what region of Kuwait is the practice where you spend most of your time located?

[INSERT DROP DOWN MENU]

1. Al-Ahmadi
2. Al Asimah
3. Farwaniyah
4. Jahra
5. Hawalli
6. Mubarak Al-Kabeer

For **Qatar** Display

In what region of Qatar is the practice where you spend most of your time located?

[INSERT DROP DOWN MENU]]

1. Al Daayen
2. Al Khor
3. Al Thakhira
4. Al Rayyan
5. Al Wakra

6. Doha
7. Madinat Al Shamal
8. Umm Slal

For **South Africa** Display

In what region of South Africa is the practice where you spend most of your time located? [INSERT DROP DOWN MENU]

1. Eastern Cape
2. Free State
3. Gauteng
4. KwaZulu-Natal
5. Limpopo
6. Mpumalanga
7. Northern Cape
8. North West
9. Western Cape

For **UAE** Display

In what region of UAE is the practice where you spend most of your time located? [INSERT DROP DOWN MENU]

1. Abu Dhabi

2. Dubai
3. Sharjah
4. Umm al-Qaiwain
5. Fujairah
6. Ra’s al-Khaimah

S6 How many years have you been in clinical practice beyond your hospital training years, residency or fellowship?

If you are still in your residency or have not been in clinical practice for at least one year, please enter “0” (zero).

Years in practice _ _ _ years

[IF IN PRACTICE ≥ 2 YEARS (S6r2+) ASK S7, ELSE TERMINATE]

S7 Do you manage type 2 diabetes patients in your clinical practice?

1. Yes
2. No

[IF MANAGING T2D PATIENTS (S7/1) CONTINUE SURVEY QUESTIONAIRE, OTHERS TERMINATE]

Healthcare Professionals Clinical Decision-Making Practices

Introduction

Diabetes is rising worldwide but it is reaching an epidemic level in the Middle East and Africa, contributing to higher healthcare expenditure, mortality and cardiovascular complications. Global recommendations for the prevention of diabetes and associated cardiovascular complications are needed to help curtail the problem of diabetes effectively.

Despite major therapeutic advances leading to improved outcomes over the past two decades, cardiovascular disease (CVD) remains the leading cause of morbidity and mortality in patients with type 2 diabetes (T2D). Over that time, the prevalence of T2D has increased, while the excess risk of adverse cardiovascular events in patients with T2D compared to patients without T2D has remained largely unchanged. Accordingly, the development of treatment strategies to improve CV outcomes in this vulnerable patient population remains a major priority.

Rational medical decision-making should not only address simple question of effectiveness and accuracy, but also a broader range of questions that take into consideration cost, availability, side effects, patients' concerns and preferences. Thus, quality clinical decision-making is a major factor underpinning quality cardiovascular outcomes in T2D, in particular for most vulnerable patient groups. Understanding of the current clinical decision-making practices is essential in developing strategies for improvement of cardiovascular outcomes. Therefore, this questionnaire is designed to assess the HCPs clinical decision-making behaviours in their management of T2D with established atherosclerotic cardiovascular disease or high cardiovascular risk.

Medicine-specific and patient factors

Q1 What are the factors that you consider when selecting an antihyperglycemic treatment for adults with T2D? *(please rank the below from 1-12, 1 as being the most important)*

Factors	***Please rank from 1-10, 1 as being the most important***
Efficacy	
Hypoglycaemia	
Weight benefit	
Cardiovascular safety	
Cardiovascular benefit	
Renal benefit	
Route of administration (e.g. oral, SC)	
Frequency of administration	
Other safety/adverse events	
Cost (direct cost of medicine)	
Affordability (ability of the patients to pay the cost of medicine)	
Access to medicine/reimbursement of medicine	

For questions Q2-Q8, please select the option that best describe your clinical decision-making practice through the 5-point rating scale:

Strongly Agree/ Agree/ Neutral/ Disagree/ Strongly Disagree

T2D Management Decisions

Q2 Which of the following *clinical factors* influence your T2D management decisions?

Factors
HbA1C
Safety profile of medications (adverse events)
Previous antidiabetic medication(s)
Concomitant medication(s)
*Atherosclerotic Cardiovascular Disease(ASCVD)** <br>*(see glossary)*
*High risk for ASCVD** (see glossary)*
Heart Failure
Chronic Kidney Disease
Obesity/overweight
Disease duration
Complications of diabetes

Q3 Which of the following *patient related factors* influence your T2D management decisions?

Factors
Age
Gender
Ethnic background
Language
Culture/ lifestyle
Level of education
Religion

Social influence (caregiver, social media, public or patient misconception, etc.)
Availability of personal/home support or assistance (family member, caregiver,etc.)
Impact on *health-related Quality of Life*** (see glossary)*
Administration preference
Treatment adherence/compliance (eg. lack of adherence)
Cost of medicines
Affordability (patient's ability to pay for the treatment)
Patient expectation for efficacy
Safety related concern(s)
Patient refusal to treatment/advice

Q4 Which of the following *practice related factors* influence your T2D management decisions?

**Factors**
Time constraints in the clinic
Influence of pharmaceutical companies
Infrastructure related factors *(cold chain requirements, distribution facilities, etc.)*
Working in private practice versus public practice
Availability of treatment in your setting
Access to other healthcare professionals<br>*(i.e. pharmacist, nurse who can support patient treatment)*
Bureaucracy in prescribing certain medications
Insurance and reimbursement criteria in your setting

Q5 Which of the following *physician related factors* influence your T2D management decisions?

Factors
International clinical guidelines
Local clinical guidelines if available
Scientific publications/updates
Education, practice, experience outside your country
Influence of key opinion leader(s)
Medical experience (years on practice)
Medical knowledge and specialization
Continuous medical education, professional development and update
Previous experience with the product

Shared Clinical Decision-making

Q6 Which of the following describe your clinical decision-making practice in terms of process?

Factors
Recognition and clarification of the problem
Identification of potential solutions
Discussing the options and uncertainties
Providing tailor-made information
Checking understanding and reactions of the patient

Checking patient’s preferences before treatment selection
Exploring the patient’s view after treatment selection
Agreeing with the patient about a course of action
Implementing the chosen course of action
Arranging follow-up with the patient

Q7 Which of the following describe your clinical decision-making practice in terms of patient empowering?

**Factors**
Treating patients as individuals
Tailor information to individual patient’s needs
Build good relationships with patients
Exchange of views with patients
Encourage patients to participate in the decision-making process
Explain the rationale for and the characteristics of proposed treatment
Address practical difficulties in medicine taking
Check patients’ understanding of what has been agreed
Check patients’ ability to follow the agreed treatment
Regularly review medications with patients’ participation
Explain to patients the outcomes and consequences of non-compliance
Discuss the impact of the disease and medication on patients’ quality of life

Q8 Which of the following strategies could improve shared decision-making, leading to improved patient and intended therapeutic outcomes?

Factors
Communication skills of physicians/healthcare professionals
Coaching and question prompt for patients
*Patient decision aids**** (see glossary)*

GLOSSARY:

***Atherosclerotic Cardiovascular Disease (ASCVD):** coronary heart disease, cerebrovascular disease, or peripheral arterial disease presumed to be of atherosclerotic origin'.[1]

Documented ASCVD can be defined either clinical or unequivocal on imaging. Documented clinical ASCVD includes previous acute myocardial infarction, acute coronary syndrome, coronary revascularization and other arterial revascularization procedures, stroke and transient ischemic attack, aortic aneurysm and peripheral arterial disease. Unequivocally documented ASCVD on imaging includes plaque on coronary angiography or carotid ultrasound or on computed tomography angiography.

****High risk for ASCVD:** patients with target organ damage[a] and/or T2D duration longer than 10 years and/or multiple ASCVD risk factors[b]

[a] Target organ damage: retinopathy, nephropathy, neuropathy, left ventricular hypertrophy

[b] ASCVD risk factors: age ≥ 55 years,[4] hypertension, dyslipidemia, smoking, obesity

***Health Related Quality of Life:** A patient's level of physical and psychosocial functional well- being as affected by disease and/or treatment.

****Patients' decision aids** are tools designed to communicate the best available evidence on treatment or screening options to patients in ways that encourage them to engage with their healthcare providers to choose an intervention that is consistent with the evidence and with their personal values. Patients' decision aids incorporate three important elements: information provision about the clinical condition and available treatment options; risk communication about the benefit and harms of every treatment option; and values clarification and guidance in deliberation and communication. Decision aids work by presenting balanced information about advantages and disadvantages of all treatment options in a way that enables patients to process this information without bias. Decision aids are designed to supplement rather than to replace the clinician-patient interaction.

Appendix C. Reimbursement status of cardioprotective medications in PACT-MEA countries.

Country	GLP-1 RAs		SGLT2is	
	Drug	Status	Drug	Status
**Bahrain**	• Victoza<br>• Trulicity<br>• Ozempic	• MOH/Government for Bahrainis only in secondary care; private: covered by insurance<br>• Private: covered by insurance<br>• Private: covered by insurance	• Forxiga<br>• Jardiance	• Private: covered by insurance<br>• MOH/Government for Bahrainis only in secondary care; private: covered by insurance
**Egypt**	All GLP-1 RAs	• Not reimbursed in national health insurance and reimbursed in private health insurance and some institutions	• Dapagliflozin<br>• Generic Dapagliflozin	• National and private health insurance<br>• Reimbursed in national health insurance
**Jordan**	• Victoza<br>• Ozempic	• Private insurance in the private sector and available and reimbursed in some institutions (public sector)<br>• Private insurance in the private sector; not available in public sector	• All SGLTis	• All are under private insurance except Canagliflozin which is not registered in Jordan, Some SGLT-2 are available and reimbursed in some institutions
**Kuwait**	• Victoza<br>• Trulicity (1.5 mg only)<br>• Bydureon<br>• Rybelsus	• MOH/Government for Kuwaitis only in secondary and primary care; private: covered by insurance<br>• MOH/Government for Kuwaitis only in secondary and primary care<br>• MOH/Government for Kuwaitis only in secondary care; private: covered by insurance<br>• Private: covered by insurance	• Forxiga<br>• Jardiance<br>• Invokana	• MOH/Government for Kuwaitis only in secondary and primary care; private: covered by insurance<br>• MOH/Government for Kuwaitis only in secondary and primary care; private: covered by insurance<br>• MOH/Government for Kuwaitis only in secondary care; private: covered by insurance

**Qatar**	• Liraglutide 1.8 mg<br>• Once weekly Semaglutide<br>• Oral Semaglutide	• Government: restricted to Qatari; private: covered by insurance<br>• Government: not enlisted, currently no access for Qatari and Expats; private: covered by insurance<br>• Government: not enlisted, currently no access for Qatari and Expats; private: covered by insurance	• Dapagliflozin<br>• Empagliflozin	• Government: No restriction to Qatari and expats at secondary care and primary care; private: covered by insurance<br>• Government: No restriction to Qatari and expats at secondary care and primary care; private: covered by insurance
**South Africa** Mostly private	• Victoza<br>• Trulicity<br>• Ozempic<br>• Byetta	• Private/no state<br>• Private/no state<br>• Private/no state<br>• Private/state buy out patients with diabetes and obesity	• Forxiga<br>• Jardiance	• Private/state/patients with diabetes and HF/on application<br>• Private access only
**United Arab Emirates** Mostly publicly covered	All classes	• Full public coverage subject to formulary availability<br>• Partial private insurance coverage (not covered for low-tier insurance)<br>• GP restrictions in some insurances	All classes	Public and/or private insurance (not covered for low-tier insurance)

Abbreviations: GLP-1 RAs, glucagon-like peptide-1 receptor agonists; GP, general practice; MOH, ministry of health; SGLT2is, sodium-glucose transport protein 2 inhibitors.
